# A New Approach for Fatigue Reliability Analysis of Thin-Walled Structures with DC-ILSSVR

**DOI:** 10.3390/ma14143967

**Published:** 2021-07-15

**Authors:** Wenyi Du, Juan Ma, Changping Dai, Peng Yue, Jean W. Zu

**Affiliations:** 1 Research Center of Applied Mechanics, School of Electro-Mechanical Engineering, Xidian University, Xi’an 710071, China; dwy690@163.com (W.D.); yiliuwo@126.com (C.D.); yuepeng0317@163.com (P.Y.); 2 School of Electro-Mechanical Engineering, Guangdong University of Petrochemical Technology, Maoming 525000, China; 3 Shaanxi Key Laboratory of Space Extreme Detection, Xidian University, Xi’an 710071, China; 4 Schaefer School of Engineering and Science, Stevens Institute of Technology, Hoboken, NJ 07030, USA; jze@stevens.edu

**Keywords:** LS-SVR, probabilistic reliability analysis, rotation matrix optimization, thin-walled pipe, distributed collaborative strategy

## Abstract

Fatigue analysis is of great significance for thin-walled structures in the spacecraft industry to ensure their service reliability during operation. Due to the complex loadings of thin-walled structures under thermal–structural–acoustic coupling conditions, the calculation cost of finite element (FE) simulations is relatively expensive. To improve the computational efficiency of dynamic reliability analysis on thin-walled structures to within acceptable accuracy, a novel probabilistic approach named DC-ILSSVR was developed, in which the rotation matrix optimization (RMO) method was used to initially search for the model parameters of least squares support vector regression (LS-SVR). The distributed collaborative (DC) strategy was then introduced to enhance the efficiency of a component suffering from multiple failure modes. Moreover, a numerical example with respect to thin-walled structures was used to validate the proposed method. The results showed that RMO performed on LS-SVR model parameters provided competitive prediction accuracy, and hence the reliability analysis efficiency of thin-walled pipe was significantly improved.

## 1. Introduction

As a common structure in the spacecraft industry, thin-walled piping has been widely used for applications such as cooling the base station on a communication satellite and avoiding overheating of charge-coupled devices on a space telescope [1,2,3,4,5]. Therefore, fatigue damage estimation of thin-walled structures is critical to guarantee operational reliability. Significantly, previous work presented a FEM model under thermal–structural–acoustic coupling conditions, which cannot cater to thin-walled open-section structures without the assumption of Euler–Bernoulli beam theory. More related details on FEM modelling can be found in [6,7]. Furthermore, the results showed that the structure experienced thermal stress caused by time-varying mean temperature, which lead to low cycle fatigue (LCF) due to the relatively large stress, as well as thermal bending moment that was mainly generated by temperature disturbance of the structure [6]. Although the small stress amplitude was generated by temperature disturbance, thermal vibration cannot be ignored as it can enhance crack growth rate and increase the cumulative fatigue damage due to multiple applied cycles superimposed on LCF [6]. Therefore, in addition to LCF, thin-walled structures can produce a high cycle fatigue (HCF) failure caused by thermal vibration. To perform a feasible and rational reliability analysis of thin-walled structures, it is necessary to take LCF and HCF into account.

Due to the uncertainties of material properties, loads, and geometric parameters, deterministic analysis cannot usually accurately predict the fatigue behavior of a structure [8,9,10,11,12]. Accordingly, moment methods such as: the first order second moment method; Monte Carlo method (MCM); surrogate model, etc., are commonly employed to obtain the fatigue reliability from a probabilistic perspective. The moment method can show the reliability relationship directly, while its accuracy is significantly affected by the nonlinearity existent in the model [13,14,15]. Although the MCM provided a higher accuracy of reliability analysis by performing many simulations, the required computing cost was extremely high [16,17]. To achieve a better balance between computational efficiency and accuracy prediction, the surrogate model can be used. This method uses a relatively small amount of computation and is often an efficient choice instead of large-scale computation.

For reliability analysis of stochastic structures, surrogate models such as: the response surface method [18,19,20]; support vector machine (SVM) [21,22,23]; kriging model [24,25,26]; artificial neural network (ANN) [16], etc., have all been employed in various industries. One efficient method with good robustness used for the approximation of nonlinear functions and small samples is, least squares support vector regression (LS-SVR), which is a widely used application in structural reliability analysis. To enhance the computational efficiency and prediction accuracy of LS-SVR it is necessary to optimize model parameters. To improve optimization, more efficient intelligent algorithms were introduced to find the parameters with fast convergence speed and high accuracy; for example: the particle swarm optimization (PSO) algorithm [27], the whale optimization algorithm (WOA) [28], and the genetic algorithm (GA) [29]. It should be note that, while searching for the model parameters these algorithms may become trapped into local optimization, which can seriously affect the LS-SVM modeling accuracy.

Based on investigations of the deficiencies of some algorithms, we attempted to develop an algorithm to find the model parameters for LS-SVM with a simpler optimization process, easier programming, and stronger ability to avoid entrapment into local optimization than the other approaches. The rotation matrix is a powerful tool that can randomly rotate a vector with a specified angle, which can be used to change the search direction of a vector. In addition, the search step length can be varied by introducing a random number and attenuation coefficient so that a new search agent can be found with better performance in both the explore and exploit stages. To satisfy the optimization requirements of LS-SVR model parameters, a novel optimization algorithm called, rotation matrix optimization (RMO) was developed with suitability for two-dimensional search space. The LS-SVR model optimized by RMO is called improved LS-SVR (ILSSVR).

To address complex and nonlinear computational puzzles with multiple failure modes or multiple variables, a distributed collaborative (DC) strategy can be utilized to achieve a higher efficiency for probabilistic fatigue assessment [30,31]. By combining these two approaches, an efficient and feasible probabilistic analysis method, named DC-ILSSVR, is presented in this paper to perform the reliability estimation of thin-walled structures, in which ILSSVR is firstly established by using RMO to find the parameters for the LS-SVR model, and then DC strategy is introduced into ILSSVR to achieve higher predicted accuracy and computational efficiency.

## 2. The Improved LS-SVR (ILSSVR) Method

Both support vector regression (SVR) and LS-SVR are efficient machine learning approaches to nonlinear function approximation with small samples and excellent robustness. However, when compared to SVR, LS-SVR can transform inequality constraints into equality constraints, which greatly facilitates the solution of Lagrange multipliers. In addition, it can be found that prediction results can be easily obtained by LS-SVM with a smaller error by tuning fewer parameters [32]. To improve computational efficiency and predicted accuracy, the RMO method is proposed to optimize the model parameters of LS-SVR.

### 2.1. LS-SVR Method

Assuming that there are *n* points of $x=[x_{1},x_{2},\cdots ,x_{n}]$ for the input variables, the corresponding outputs *y* can be written according to the LS-SVR theory [33]:

(1) $$y(x)=\sum_{i=1}^{l}\alpha_{i}\psi \left(x,x_{i}\right)\;+\;b$$

where $\alpha_{i}$ are the Lagrange multipliers, $\psi \left(x,x_{i}\right)$ is the kernel function, and *b* is the bias term. α and *b* are obtained by the following equations [34]:

(2) $$\left(\begin{array}{cc} 0 & v^{T}\\ v & K+\frac{I}{\gamma } \end{array}\right)\left(\begin{array}{l} b\\ \alpha \end{array}\right)=\left(\begin{array}{l} 0\\ y \end{array}\right)$$

where ***K*** is the kernel matrix, ***v*** is a *n* × 1 vector in which the value of each element is equal to 1, and γ is the regularization parameter affecting the balance between the minimization of training error and the smoothness of the regression curve.

Radical basis function in LS-SVM model can be written as:

(3) $$\psi \left(x,x_{i}\right)\;=\exp\left(-\frac{{\left\|x-x_{i}\right\|}^{2}}{2\sigma^{2}}\right)$$

where $\sigma^{2}$ is the square width of the kernel function. For the LS-SVM model, two parameters $\sigma^{2}$ in Equation (3) and γ in Equation (2) need to be optimized to obtain the optimal model parameters.

### 2.2. Rotation Matrix Optimization Algorithm

In recent years, with the widespread application of intelligent algorithms in practical engineering, more swarm intelligence algorithms have been developed by scholars. These are based on the observations of various animals to provide heuristic ideas for optimization problems. The representative algorithms are shown in Table 1.

**Figure 1:**
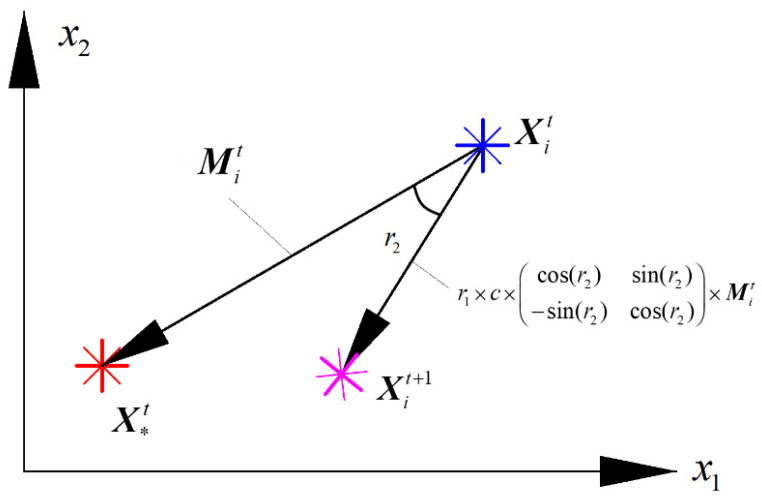
The evolution process of RMO.

Through investigations on the performance of the algorithms in Table 1, it can be found that all of them were required to update the position of search agent *i* in the search space with the help of vector $M_{i}^{t}$, in which $M_{i}^{t}$ was the vector from the present position of search agent $X_{i}^{t}$ toward the best position of all search agents $X_{*}^{t}$ at current iteration *t*. For the algorithms listed in Table 1, random variables are always introduced to update the current position $X_{i}^{t}$ of search agent with vector $M_{i}^{t}$, while the direction of vector $M_{i}^{t}$ cannot be changed, which would reduce improvement of the local search ability. Inspired by the idea of a rotation matrix, vector $M_{i}^{t}$ can be rotated and scaled as required by introducing an expansion coefficient $r_{1}$ and rotation angle $r_{2}$. In view of this, the rotation matrix optimization algorithm is proposed in this section to deal with the issue. Figure 1 shows the evolution process of rotation matrix optimization.

As was described above, there are two model parameters of LS-SVM ($\sigma^{2}\;\mathrm{and}\;\gamma$) which need to be optimized. The RMO for two dimensions of the search space can be expressed as:

(4) $$X_{i}^{t+1}=X_{i}^{t}+r_{1}\times c\times \left(\begin{array}{cc} \cos(r_{2}) & \sin(r_{2})\\ -\sin(r_{2}) & \cos(r_{2}) \end{array}\right)\times M_{i}^{t}$$

where the vector $M_{i}^{t}$ is expressed as:

(5) $$M_{i}^{t}=X_{*}^{t}-X_{i}^{t}$$

$r_{1}$ and $r_{2}$ respectively the expansion coefficient and rotation angle of $M_{i}^{t}$; c is the attenuation coefficient that declines linearly from a user defined constant scale to 0 during the iterative process, which can be written by:

(6) $$c=scale-\left(t\cdot \left(scale/Max_{iteration}\right)\right)$$

In this study, we let *c* be equal to 2; $r_{1}$ is a random variable with uniform distribution between [0, 2]; $r_{2}$ obeys the normal distribution with mean value of 0 and standard deviation of $\frac{\pi }{3}$

## 3. DC-ILSSVR Approach—Thin-Walled Tube Reliability Analysis

In this section, the DC-ILSSVR method is proposed based on the integration of ILSSVR and DC to reduce the complexity and enhance the computational efficiency of fatigue reliability estimation of thin-walled circular tube structures.

### 3.1. DC-ILSSVR Method

**Figure 2:**
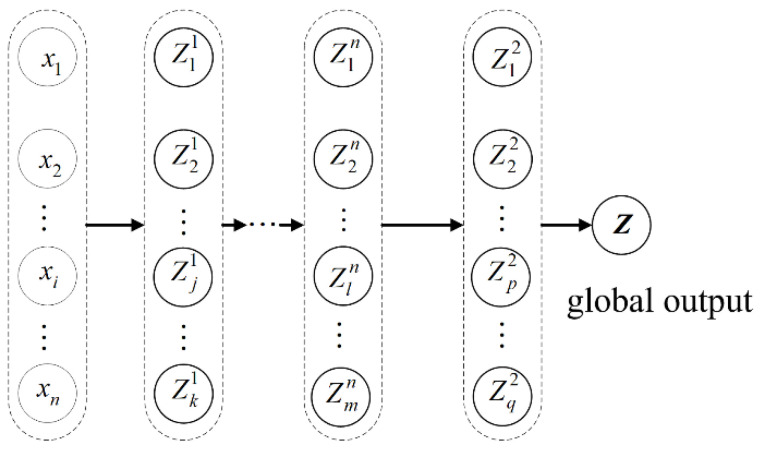
The basic framework of DC.

The DC strategy divides the complex and nonlinear problem into several simple ones, which can be solved with higher computational efficiency, and then subsequently through cooperation among the subproblems [43]. Firstly, the input variables and output responses (distributed response) of each subproblem need to be defined. Then, the distributed response should be analyzed level by level. The output response of the first level is regarded as the input variable to the second level. In this way, all distributed responses can be obtained. The output response of the last level is termed the global output response (also named collaborative response), and Figure 2 shows the basic framework of DC.

Extreme response surface method (ERSM) was developed to improve the performance of RSM with high efficiency and accuracy [19,20]. Moreover, systems in practical engineering usually contain multiple components and multiple failure modes. Suppose there are *m* components and *n* failure modes, and the random input vector of the *i*th component in the *j*th failure mode is ***X****^ij^* and the corresponding response *Y^ij^*. According to ERSM, the new response y can be obtained by the maximum value *Y^ij^*

(7) $$y=\left\{Y_{\max}^{ij},\;j\in Z_{+}\right\}$$

Furthermore, the relationship between response y and random input vector ***X****^ij^* is expressed as follows:

(8) $$y=f\left(X\right)\;=\;\left\{Y_{\max}^{ij}\left(X^{ij}\right),i=1,2,\cdots ,m,j=1,2,\cdots ,n\right\}$$

Rewrite Equation (8) by the response surface function:

(9) $$y=A_{0}+BX+X^{T}CX$$

Then DC-ERSM method for complex mechanical systems with multiple components and multiple failure modes can be established as follows:

(10) $$\left\{\begin{array}{ll} y_{\max}^{11}= & A_{0}^{11}+B^{11}X^{11}+{\left(X^{11}\right)}^{T}C^{11}X^{11}\\ y_{\max}^{12}= & A_{0}^{12}+B^{12}X^{12}+{\left(X^{12}\right)}^{T}C^{12}X^{12}\\ & \vdots \\ y_{\max}^{ij}= & A_{0}^{ij}+B^{ij}X^{ij}+{\left(X^{ij}\right)}^{T}C^{ij}X^{ij} \end{array}\right.$$

where $A_{0}^{ij}$ is the constant term under the *i*th component in *j*th failure mode, ***B****^ij^* is the linear term, and ***C****^ij^* is the quadratic term. ***B****^ij^* and ***C****^ij^* can be expressed as follows:

(11) $$B^{ij}=\left[b_{1}^{ij}\;b_{2}^{ij}\;\cdots \;b_{q}^{ij}\right]$$

(12) $$C^{ij}=\left[\begin{array}{cccc} c_{11}^{ij} & c_{12}^{ij} & \cdots & c_{1q}^{ij}\\ c_{21}^{ij} & c_{22}^{ij} & \cdots & c_{2q}^{ij}\\ \vdots & \vdots & \ddots & \vdots \\ c_{q1}^{ij} & c_{q2}^{ij} & \cdots & c_{qq}^{ij} \end{array}\right]$$

Similarly, the proposed DC-ILSSVR method is developed by combining DC strategy with ILSSVR based on the idea of extremum response. Assuming that the random input variables and corresponding responses of the *j*th component in *k*th failure mode are ***x****^jk^* and *y^jk^*, and then a new response set $Y=\left\{y_{\max}^{ij},\;i,j\in Z_{+}\right\}$ composed of the maximum values of responses is derived as follows:

(13) $$\left\{\begin{array}{ll} y_{\max}^{11} & =f\left(x^{11}\right)\;=\sum_{i=1}^{m}\alpha_{i}\psi \left(x^{11},x_{i}\right)+\;b\\ y_{\max}^{12} & =f\left(x^{12}\right)\;=\sum_{i=1}^{m}\alpha_{i}\psi \left(x^{12},x_{i}\right)+\;b\\ & \vdots \\ y_{\max}^{jk} & =f\left(x^{jk}\right)\;=\sum_{i=1}^{m}\alpha_{i}\psi \left(x^{jk},x_{i}\right)\;+\;b \end{array}\right.$$

### 3.2. Limit State Function with Strength Degradation

As mentioned above, LCF is mainly caused by time-varying mean temperature due to the relatively large stress amplitude at the fixed end. Therefore, the SWT model is chosen to express the relationship between low cycle stress and its corresponding fatigue life. This model has improved performance compared to classical strain-life methods like Coffin–Manson formula [44]:

(14) $$\sigma_{\max}\varepsilon_{L}=\sigma_{\max}\frac{\Delta \varepsilon }{2}=\frac{{\left({\sigma^{\prime }}_{f}\right)}^{2}}{E}{\left(2N_{L}\right)}^{2b}+{\varepsilon^{\prime }}_{f}{\sigma^{\prime }}_{f}{\left(2N_{L}\right)}^{b+c}$$

where $\sigma_{\max}$ is the maximum value of low cycle stress, $\varepsilon_{L}$ is the corresponding strain, *E* is elastic modulus, ${\sigma^{\prime }}_{f}$ and ${\varepsilon^{\prime }}_{f}$ are fatigue strength coefficient and fatigue ductility coefficient, $N_{L}$ is the number of cycles to failure under low cycle fatigue loading, and *b* and *c* are fatigue strength exponent and fatigue ductility exponent.

The damage caused by one cycle of low cycle loading is expressed by [45]:

(15) $$D\left(L1\right)=\frac{1}{N_{L}}$$

On the other hand, small stress generated by disturbance temperature of thin-walled structures leads to HCF failure. In other words, the structure experienced a complex loading with numerous HCF cycles superposed on per LCF loading. Generally, an S-N curve can be used to calculate the HCF life corresponding to the stress amplitude. As one of the most used approaches, Miner’s rule was employed to calculate the cumulative damage of thin-walled structure caused by high cycle loadings [6,46]. However, the nonlinearity and coupled effect between HCF and LCF during fatigue process were ignored. In view of this, the HCF damage in one LCF cycle of the structure is presented to address the HCF-LCF interaction based on previous work [47]:

(16) $$D\left(H1\right)=\sum_{i=1}^{m}\frac{n_{Hi}}{N_{Hi}\log{\left(N_{Hi}\right)}^{-\alpha_{eq}}}$$

where *N_Hi_* is the number of cycles to failure of high cycle stress $\sigma_{Hi}$; *n*_Hi_ is the number of applied cycles in a low cycle loading; $\alpha_{eq}$ is the ratio of high and low stress range.

Combined with Equations (15) and (16), the total fatigue damage of one cycle caused by low and high cycle loading can be obtained as follows:

(17) $$D\left(1\right)=D\left(L1\right)+D\left(H1\right)=\frac{1}{N_{L}}+\sum_{1=1}^{m}\frac{n_{Hi}}{N_{Hi}\log{\left(N_{Hi}\right)}^{-\alpha_{eq}}}$$

Thus, the cumulative fatigue damage of *n* cycles can be calculated by:

(18) $$D\left(n\right)=nD\left(1\right)=n\left[D\left(H1\right)+D\left(L1\right)\right]$$

According to the residual strength theory, the residual strength of the structure can be written as follows:

(19) $$R\left(n\right)=R\left(0\right){\left[1-D\left(n\right)\right]}^{a}=R\left(0\right){\left[1-nD\left(1\right)\right]}^{a}$$

where *R*(0) is the initial static strength of the structure and *a* is the strength degradation coefficient of the material.

Then, the limit state function can be expressed by the difference between remaining strength and the working load:

(20) $$g\left(x\right)=R\left(n\right)\;-\;\sigma_{0}=R\left(0\right){\left[1-nD\left(1\right)\right]}^{a}-\sigma_{0}$$

where $\sigma_{0}$ is the external load. If *g*(*x*) > 0, the structure is safe; otherwise, the structure is a failure.

### 3.3. Reliability Analysis Procedure Based on DC-ILSSVR

In this section, based on DC-ILSSVR, a probabilistic fatigue analysis procedure is proposed to carry out the reliability estimation of random thin-walled tube. The steps are as follows:

(1) Based on 3*σ* principle, 100 groups of samples of random input variables were firstly generated with Latin hypercube sampling (LHS). According to the previous work, the finite element model of thin-walled tube structure under thermal–structural–acoustic coupling can be expressed by Equation (21). More details can be found in reference [6]. Then, 100 groups of dynamic response samples can be obtained by substituting the 100 groups of input samples into the finite element model:

(21) $$\left\{\begin{array}{l} M_{S}\ddot{U}+C_{S}\dot{U}+K_{S}U=F_{T}+F_{f}+F_{S}\\ C_{T}{\dot{T}}_{0}+K_{T0}T_{0}=Q_{0}+C_{TS}-R_{T0}\\ C_{T}{\dot{T}}_{1}+\left(K_{T1}+R_{T1}\right)T_{1}=\frac{2}{\pi }\left(Q_{0}+C_{TS}\right)\\ M_{f}\ddot{P}+C_{f}\dot{P}+K_{f}P+R_{Sf}\ddot{U}=0 \end{array}\right.$$

(2) The 100 groups of samples in step (1) are randomly divided into a training set of 70 samples and a testing set of 30 samples. Then, the ILSSVR surrogate model is trained and verified by the training. The testing sets go through the same process;
(3) A total of 10,000 groups of input samples are generated by MCM, and then the corresponding 10,000 groups of output responses are obtained by using the ILSSVR surrogate model built in step (2);
(4) The output responses in step (3) are regarded as the input samples of second level to obtain the corresponding *N_L_* by Equation (14);
(5) Similarly, the samples of *N_H_* under 10,000 simulations are predicted with same input variables;
(6) The output responses of *N_L_* and *N_H_* are taken as the input samples of the third level, and cumulative fatigue damage *D*(*n*) can be obtained by Equations (15)–(18);
(7) Take *D*(*n*) as the input samples of the fourth level to get *R*(*n*) by using Equation (19), and *R*(*n*) as the input of the fifth level to gain the limit state function *g*(*x_i_*) by Equation (20);
(8) The reliability of the structure *R_r_* can be approximately obtained by recording the number of samples *m* when *g*(*x_i_*) > 0.

(22) $$R_{r}=m/10000$$

## 4. Case Study

**Figure 3:**
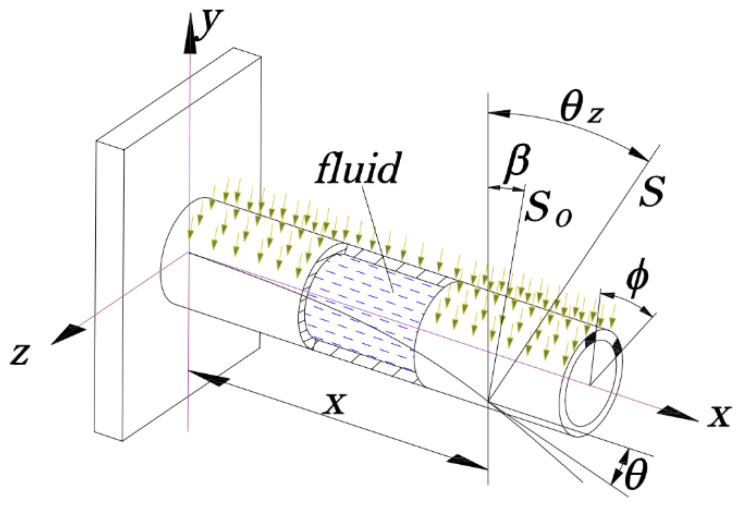
The thin-walled tube structure under thermal–structural–acoustic coupling.

In this paper, the thin-walled tube structure under thermal–structural–acoustic coupling condition is selected as the research subject, as shown in Figure 3. From Figure 3, *θ* is the rotation angle of cross section; *θ_z_* is the angle between *y* axis and the external normal of the structural exterior surface after tube deforming; *S*_0_ is the heat flux of solar radiation; *S* is the net radiation heat flux exerted on the structural surface; *β* and ϕ are, respectively, the incident angle of sunlight, and circumferential angle of cross section. More details and model parameters can be referred to in [6].

**Figure 4:**
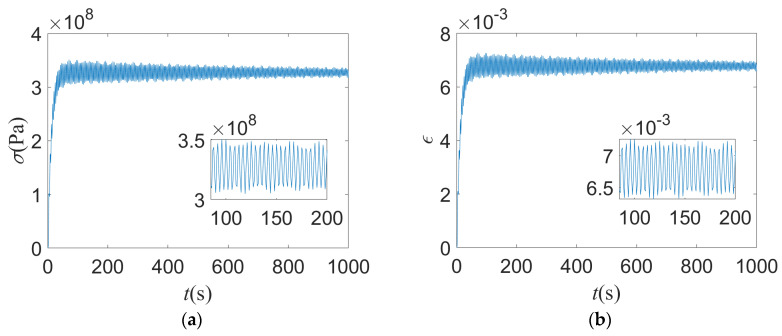
The dynamic response of of maximum stress (**a**) and strain (**b**) of deterministic structure.

For deterministic structure of given model parameters, the maximum stress and strain of the structure occur at the fixed end of the tube. Based on the established finite element model of thin-walled tube structures under thermal–structural–acoustic coupling, the dynamic response of maximum stress and strain at fixed end is shown in Figure 4.

The deterministic method cannot model the dynamic response of the structure well due to the randomness of material parameters, geometry sizes, and working loads. In view of this, to investigate the influence of the uncertainties on the dynamic reliability of the structure, the size parameters, such as the length of tube *l*, the inner radius *r*, and the wall thickness *t*, as well as material parameters, such as elastic modulus *E* and density *ρ* are selected as random variables to extract the input samples by LHS. In this section, 100 sets of input vectors [*l*, *r*, *t*, *E*, *ρ*] are generated, and the corresponding output responses can be obtained to build the DC-ILSSVR model based on the constructed FE model in previous work [6], in which the stress and strain at the fixed end are the maximum value of dynamic responses that occur at the thin-walled tube structure.

**Figure 5:**
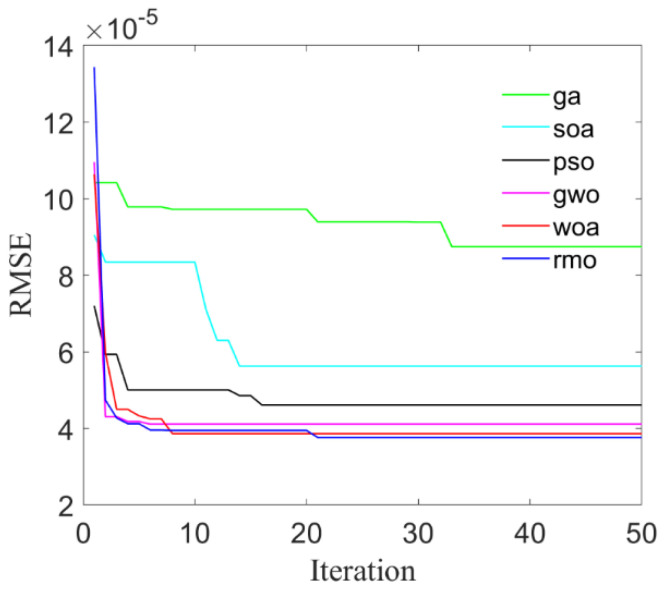
The convergence curves of maximum stress.

**Figure 6:**
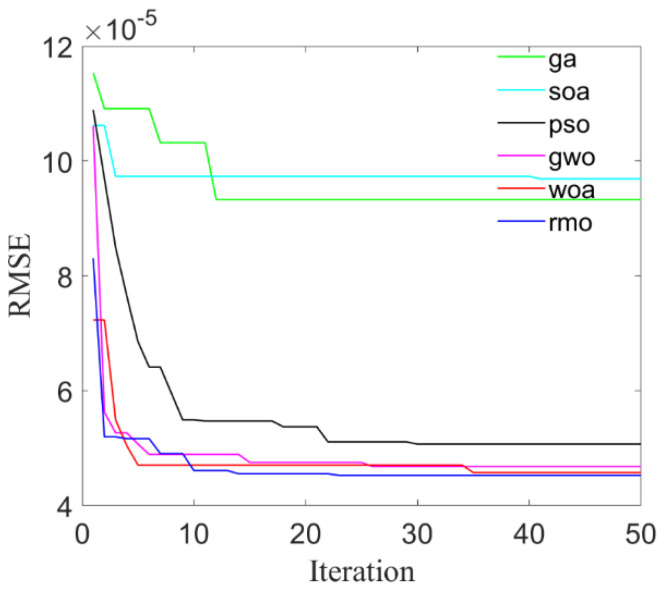
The convergence curves of maximum strain.

To validate the proposed optimization approach, six kinds of intelligent algorithm including: genetic algorithm (GA); seagull optimization algorithm (SOA); particle swarm optimization (PSO); gray wolf optimization (GWO); whale optimization algorithm (WOA); and RMO are compared to search for the optimal parameters to be used in LS-SVR modeling. Root Mean Square Error (RMSE) is used as the fitness function of the six optimization algorithms, and the convergence curves with respect to maximum stress and maximum strain are shown in Figure 5 and Figure 6, respectively.

These six algorithms mentioned above are used to perform 100 times of optimization on LS-SVR (the prediction of maximum stress) to calculate the mean and standard deviation. The optimal fitness, mean value, and standard deviation of fitness, as well as running time, are compared to verify the proposed method, and the results are shown in Table 2.

**Figure 7:**
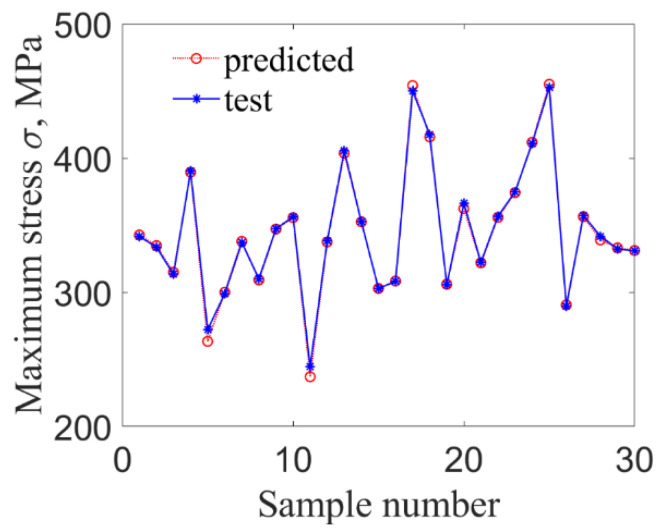
The maximum stress at fixed end.

**Figure 8:**
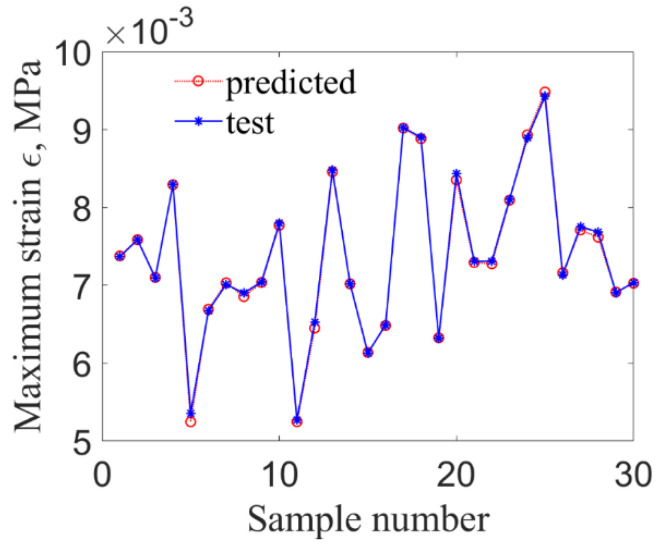
The maximum strain at fixed end.

Compared with other algorithms, RMO has a better performance in finding the optimal solutions of LS-SVR model and convergence speed. Accordingly, the LS-SVR model optimized by RMO is used to predict the maximum stress and strain based on the extracted samples, in which 30 groups of inputs samples are selected randomly as test sets. The prediction results by the proposed method are shown in Figure 7 and Figure 8.

**Figure 9:**
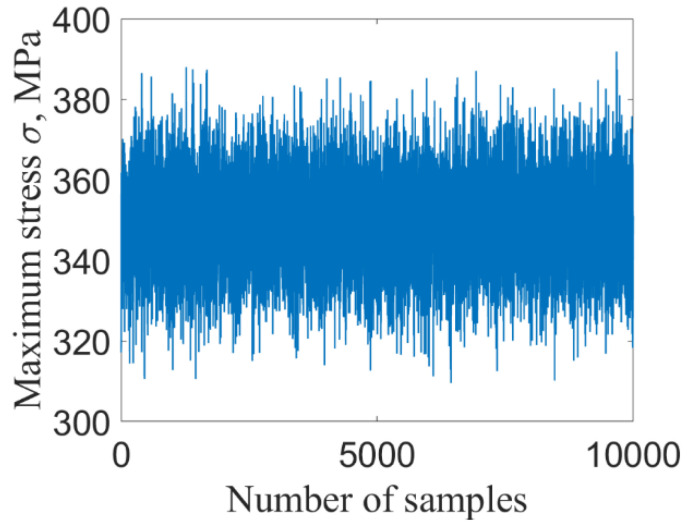
Stress.

**Figure 10:**
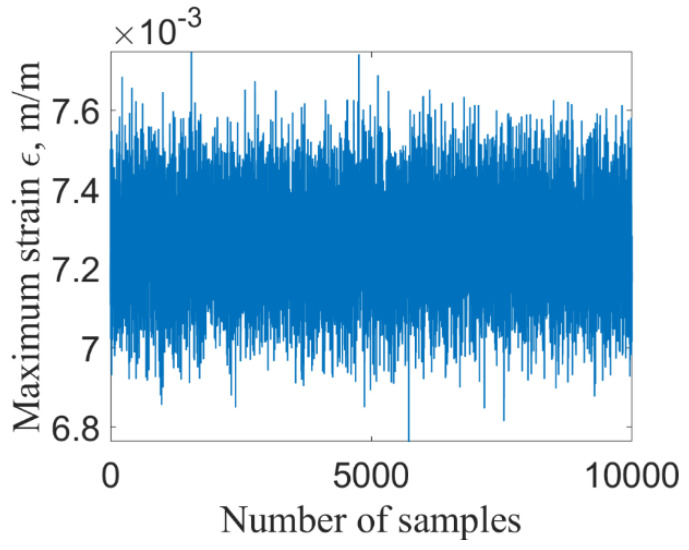
Strain.

**Figure 11:**
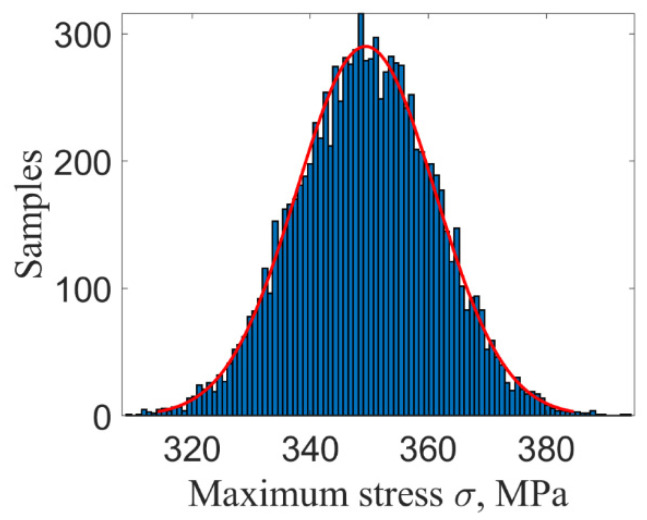
Distribution histogram of stress.

**Figure 12:**
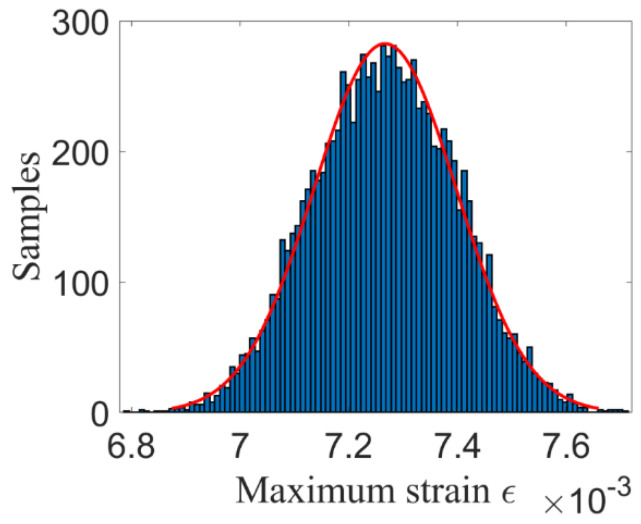
Distribution histogram of strain.

Figure 9 and Figure 10 show the maximum stress and strain by using ILSSVR surrogate under 10,000 groups of input samples, respectively. Figure 11 and Figure 12 are the distribution histogram of maximum stress, and strain, and the maximum stress. Strain obeys the normal distribution of $N\left(3.5123\times 10^{2},\;{\left(1.0981\times 10^{1}\right)}^{2}\right)$ and $N\left(0.0073,\;{\left(9.7325\times 10^{-5}\right)}^{2}\right)$

**Figure 13:**
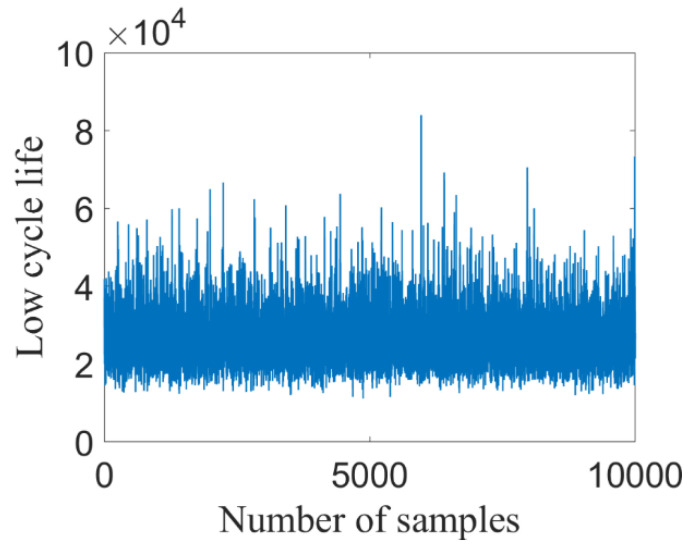
Simulated samples of *N_L_*.

**Figure 14:**
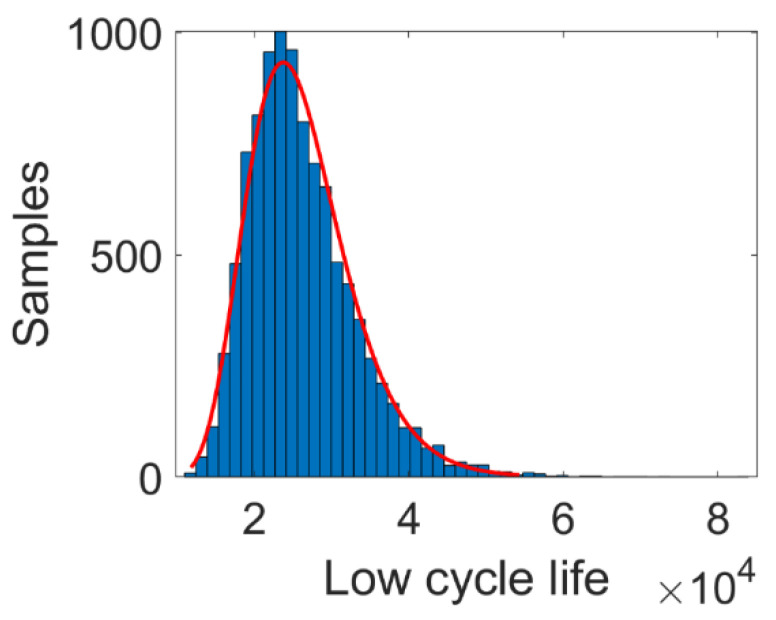
Distribution histogram of *N_L_*.

According to the simulated maximum stress and strain, the low cycle fatigue life can be calculated by SWT model. The parameters of SWT model are as follows: ${\sigma^{\prime }}_{f}=1755.94$ MPa, ${\varepsilon^{\prime }}_{f}=1.6115$, b=−0.0859, c=−0.7712. Figure 13 presents the predicted results of *N_L_* under 10,000 sets of samples, and Figure 14 presents the corresponding distribution histogram. Note from Figure 14 that *N_L_* follows lognormal distribution, in which the red line is the probabilistic density function.

**Figure 15:**
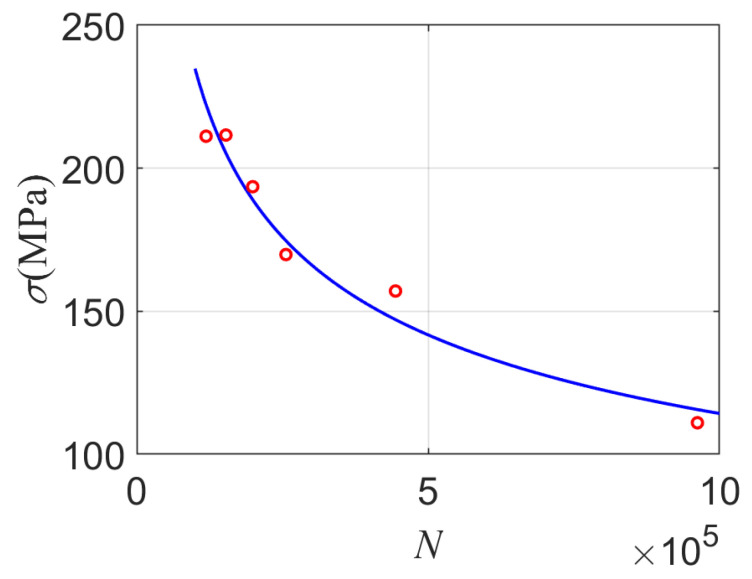
S-N curve of thin-walled tube pipe material.

The fatigue test data of thin-walled tube material relating to stress, and its fatigue life is shown in Table 3. According to the six groups of data in Table 3, the S-N curve of the material can be fitted, as shown in Figure 15.

On the other hand, the high cycle loadings caused by disturbance temperature can be approximately obtained by the dynamic response as follows: $\sigma_{a1}=23$ MPa, $\sigma_{a2}=24$ MPa, $\sigma_{a3}=25$ MPa. Because the high cycle stress is not symmetrically cyclic, the equivalent fully reversed stress can be achieved by using mean stress correction, and then the corresponding high cycle life is accordingly obtained by using the *S-N* curve.

In this work, the number of cycles corresponding to high cycle stress determined by the rain-flow counting method is 200, and the Goodman mean stress correlation is used to convert the high cycle stress into the corresponding equivalent stress under zero mean stress conditions.

The tensile strength of the given material is 480 MPa, and the corresponding equivalent stress under symmetrical cycle fatigue loadings is, respectively, $\sigma_{a1}^{-1}=78.86$ MPa, $\sigma_{a2}^{-1}=82.89$ MPa, $\sigma_{a3}^{-1}=85.71$ MPa. The high cycle fatigue life corresponding to high cycle stresses can be obtained by using the S-N curve in Figure 15.

**Figure 16:**
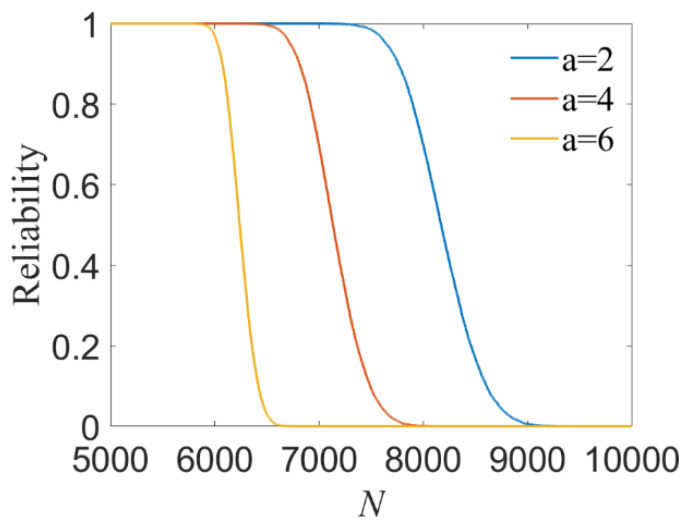
Relationship between reliability and *N* with different *a*.

Based on the simulations with respect to fatigue life under low cycle loading and high cycle loading, the dynamic reliability of random thin-walled tube structure can be obtained by the strength degradation formula. Figure 16 shows the variation of reliability with different degradation coefficient *a*.

From Figure 16 and Table 4, it can be found that the reliability with various *a* is in line with that presented in the previous work [6]. Moreover, the results indicate that the DC-ILSSVR method offers an effective and feasible probabilistic fatigue analysis approach, with a significant improvement in computational efficiency and predicted accuracy.

## 5. Conclusions

In this study, a new probabilistic reliability analysis approach for thin-wall structures under thermal–structural–acoustic coupling was established based on the integration of LS-SVR, RMO, and DC strategies. The main achievements are as follows:

(1) A new optimization algorithm named RMO was developed to provide a linear combination of the position of current search agent and best fit search agent, which can avoid the local optimization with simple calculation;
(2) The LS-SVR model with the optimal parameters developed by employing RMO was named as ILSSVR, and the results indicated that the method provided fast convergence speed, and better prediction accuracy than other methods used in LS-SVR modeling;
(3) The DC-ILSSVR proved an effective and feasible fatigue analysis approach by embedding DC strategy into the ILSSVR surrogate model to establish a probabilistic reliability assessment procedure of thin-wall structures.

## Figures and Tables

**Table 1:** Swarm intelligence algorithms.

Algorithms	Year
Sparrow search algorithm (SSA) [35]	2020
Butterfly optimization algorithm (BOA) [36]	2019
Coyote optimization algorithm (COA) [37]	2018
Seagull optimization algorithm (SOA) [38]	2018
Whale optimization algorithm (WOA) [39]	2016
Sine cosine algorithm (SCA) [40]	2016
Elephant herding optimization (EHA) [41]	2016
Bat-inspired algorithm (BA) [42]	2010

**Table 2:** The optimized performance of the 6 methods in 100 simulations.

Method	Best Fitness (10^−5^)	Mean Value (10^−5^)	Standard Deviation (10^−5^)	Computing Time (s)
GA	4.125	4.477	0.232	4238
SOA	3.569	4.129	0.201	4332
PSO	3.471	4.022	0.167	4021
GWO	3.393	3.875	0.118	4450
WOA	3.447	3.926	0.120	4234
RMO	3.379	3.776	0.113	3970

Optimization configuration: number of search agent: 40, the maximum iteration: 50 Computer configuration: 16GB RAM, intel i5-11300H CPU. Algorithm parameter is as follows: 1. GA: The gene length of single trait was 10, the variation rate was 0.05, and the crossover rate was 0.8; 2. SOA: fc = 2, u = 1, v = 1; 3. PSO: The learning factors 1 and 2 are both equal to 1.5, and the maximum and minimum inertia weights are 0.8, and 0.4, respectively.

**Table 3:** Fatigue test data of materials.

Load (MPa)	111.011	157.071	169.824	193.543	211.600	211.189
Life (×10^5^)	9.62	4.44	2.56	1.99	1.53	1.19

**Table 4:** Reliability, and calculation time of different methods.

	MCM	RFM [6]	DC-ERSM	DC-ILSSVR
Reliability	96.26%	97.96%	97.81%	97.06%
Calculation time (s)	91491	1532	1681	553

Note: RFM denotes random factor method.

## Data Availability

Some or all data, models, or code that support the findings of this study are available from the corresponding author upon reasonable request.
